# Supplementary figures and images for: Topological defects in self-assembled patterns of mesenchymal stromal cells in vitro are predictive attributes of condensation and chondrogenesis
Source: PLoS One. 2024 Mar 28;19(3):e0297769. doi: 10.1371/journal.pone.0297769 (PMC10977694; doi:10.1371/journal.pone.0297769)

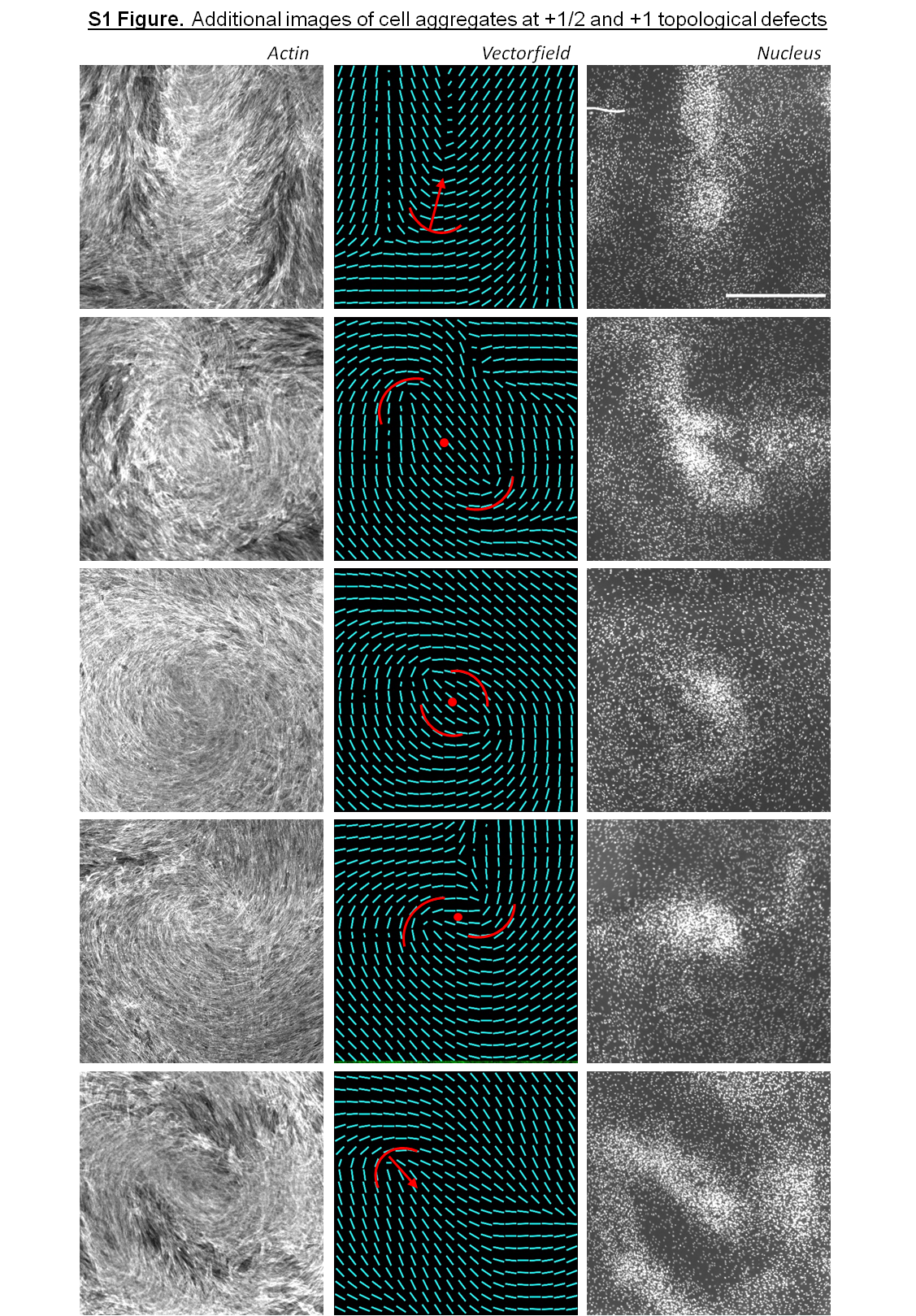

Supplement: S1 Fig — Five regions (cropped from whole-well stitched images) showing that cells aggregate at sites of +1/2 and +1 topological defects. The aggregates are visible in the nucleus images, while the defects are visible in the actin and corresponding orientation vectorfield images. Orientation vectorfields were generated using the OrientationJ plugin in ImageJ (see Methods). Scale bar 1000 μm. (TIF) [file pone.0297769.s001.tif]

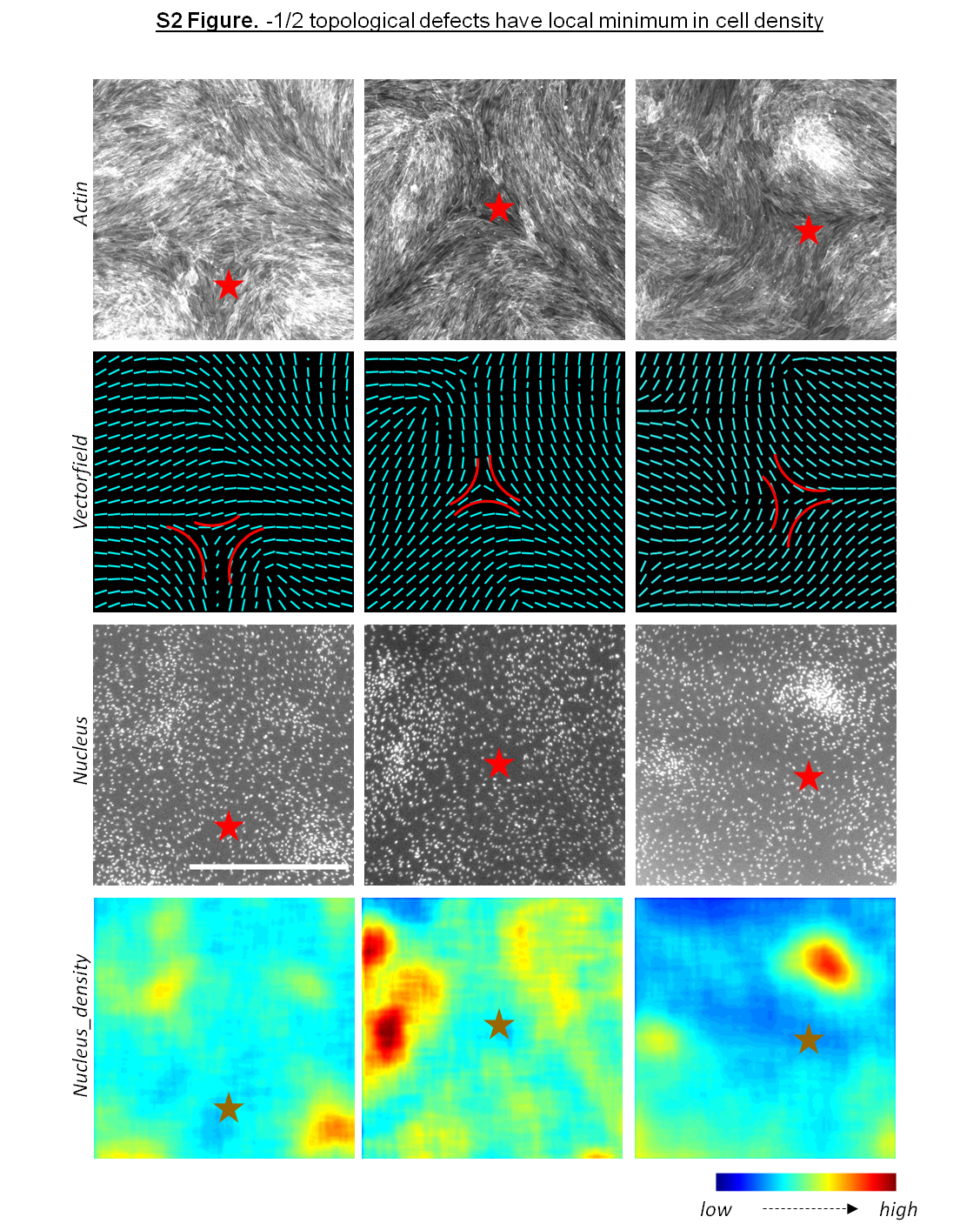

Supplement: S2 Fig — Three regions (cropped from whole-well stitched images) showing that cells recede from -1/2 topological defects. The defects are visible in the actin and corresponding orientation vectorfield images, while the density-minima are visible in the nucleus density colormap. Orientation vectorfields were generated using the OrientationJ plugin in ImageJ (see Methods). Nucleus density colormaps were generated using an averaging filter (100 pixels, i.e., 16 μm) on the nucleus intensity images in MATLAB. Scale bar 1000 μm. (TIF) [file pone.0297769.s002.tif]

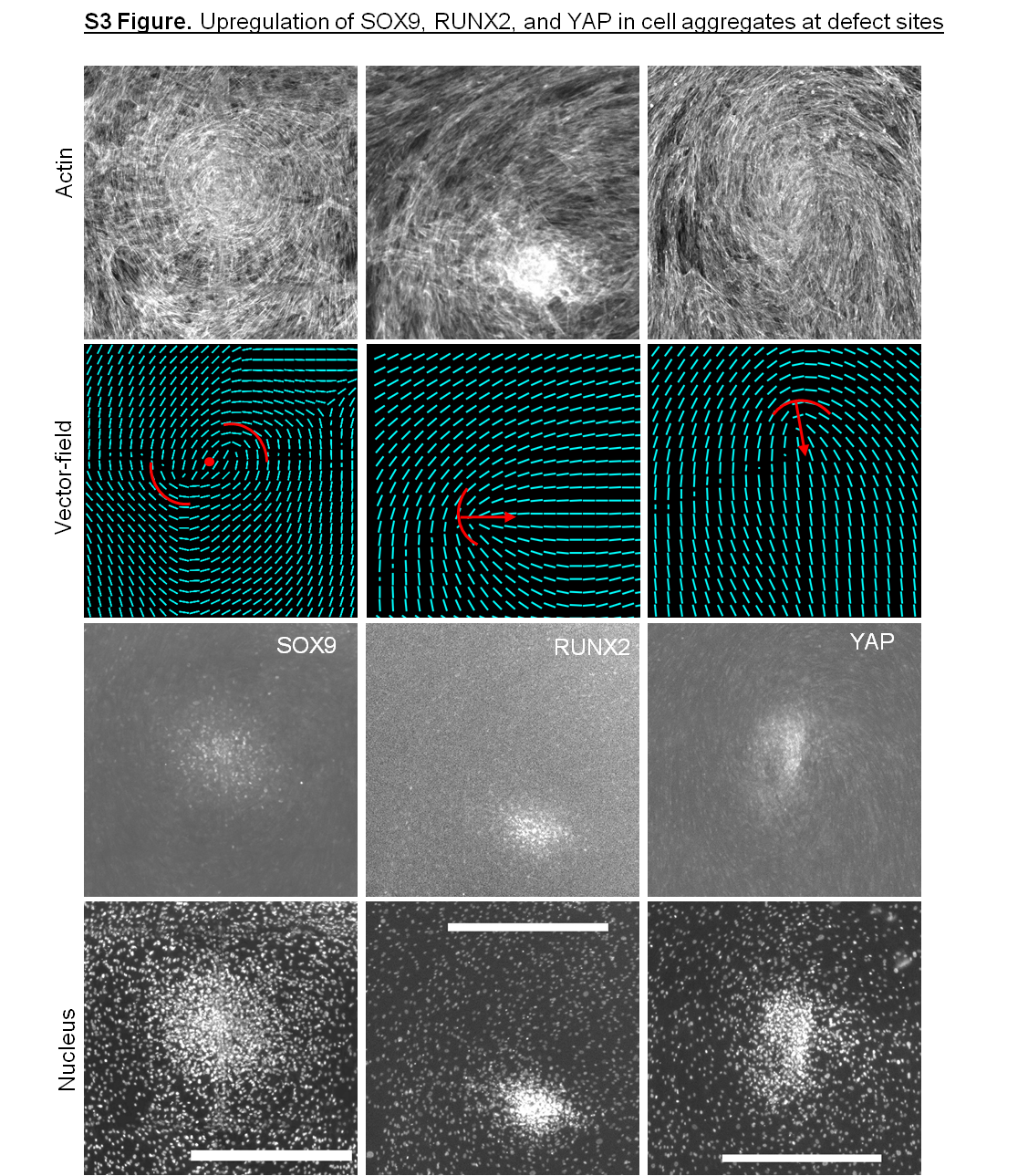

Supplement: S3 Fig — Regions cropped from whole-well stitched actin, nucleus, and transcription factor images show higher levels of SOX9, RUNX2, and YAP in the nuclei of cells aggregated at the topological defect sites. SOX9, RUNX2, and YAP were stained in day 10, day 9, and day 8 samples respectively. Scale bar 1000 μm. (TIF) [file pone.0297769.s003.tif]

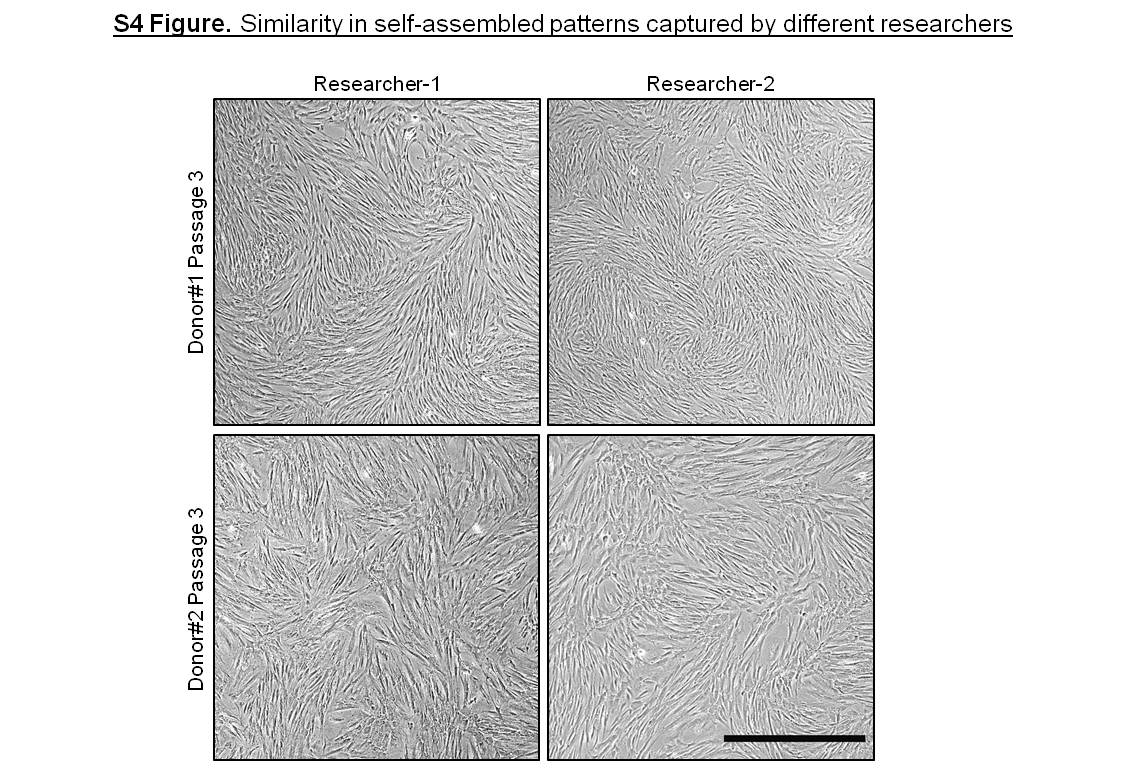

Supplement: S4 Fig — Phase-contrast images of bm-MSCs at confluency captured by two different researchers on day 10–12 post seeding with 1500–2000 cells/cm2 starting density. Scale bar 1000 μm. (TIF) [file pone.0297769.s004.tif]

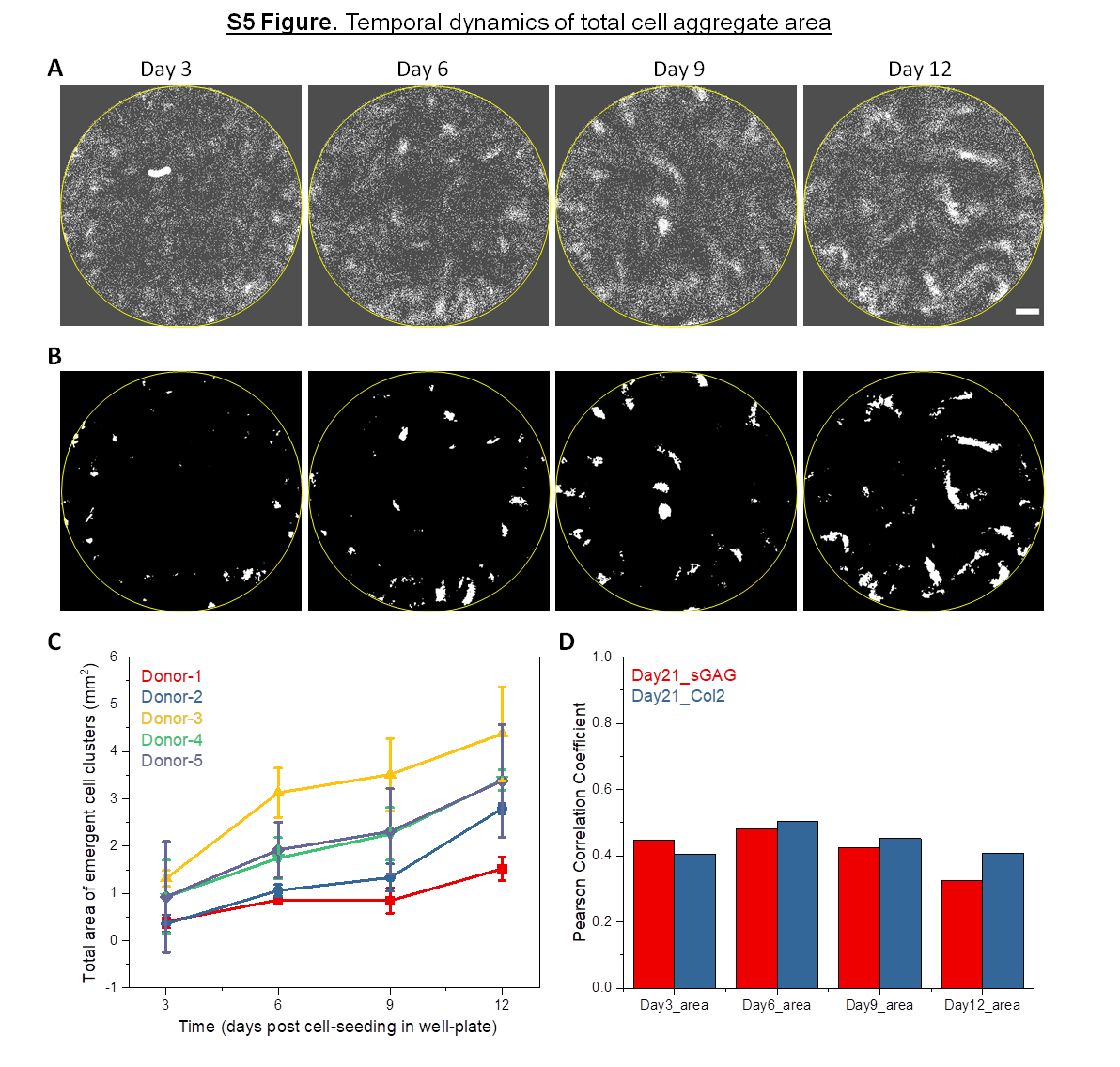

Supplement: S5 Fig — (A) Whole-well stitched nuclei images from a single donor at time 3, 6, 9, 12 days post seeding. (B) Binary masks generated by thresholding the above nuclei images. Spots corresponding to debris were manually removed. (C) Total cell aggregate area per well plotted as a function of time for 5 MSC donors (n = 3 technical replicates per donor). (D) Pearson correlation coefficient for day 3, 6, 9, 12 total area vs levels of matrix protein, n = 5 donors. Scale bar 1000 μm. (TIF) [file pone.0297769.s005.tif]

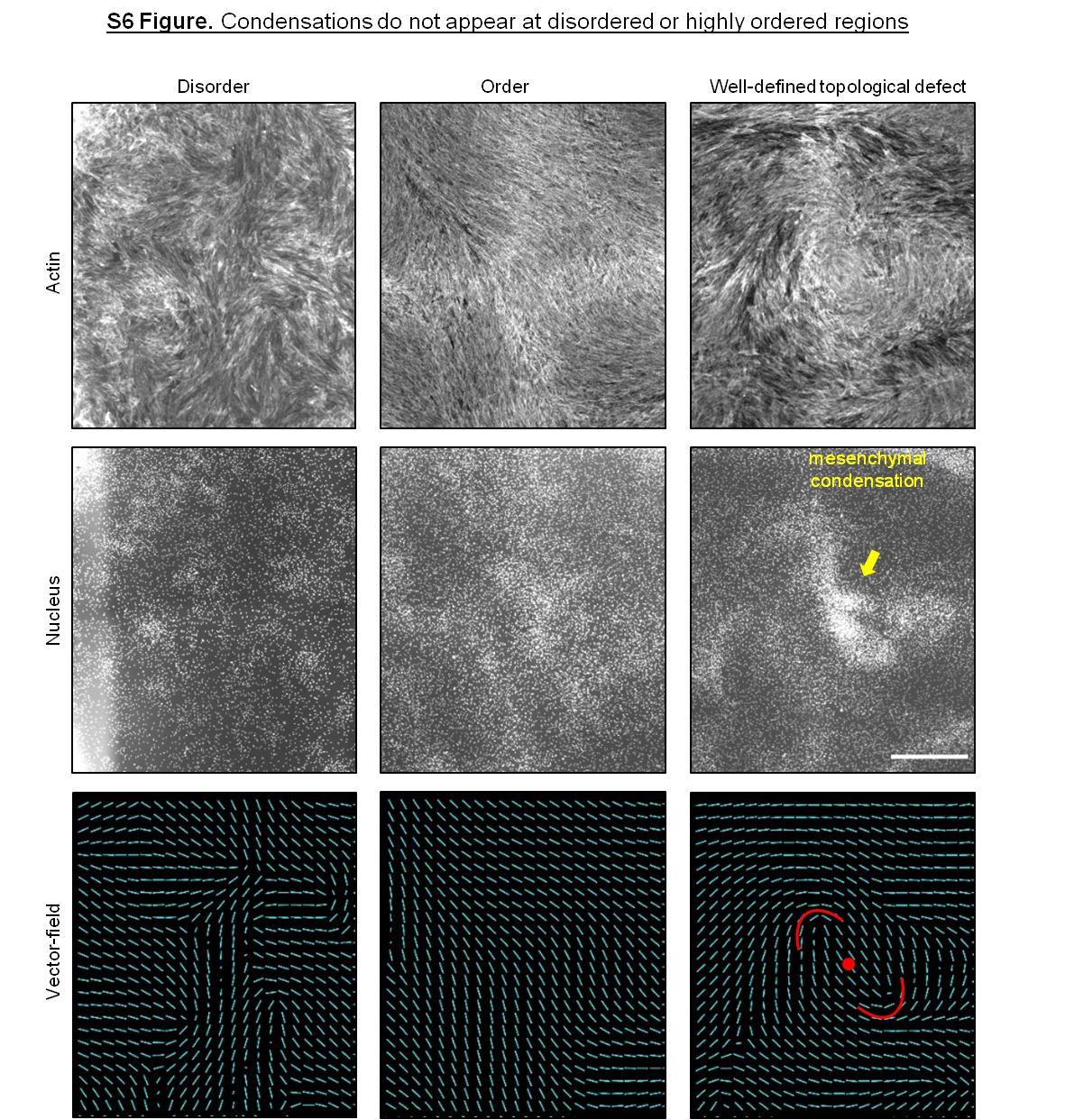

Supplement: S6 Fig — The first row shows actin images corresponding to three regions where the pattern may be classified as having ‘disorder’, ‘order’, and ‘topological defect’. The second and the third rows show the corresponding nucleus and vectorfield images for the regions. Scale bar 1000 μm. (TIF) [file pone.0297769.s006.tif]

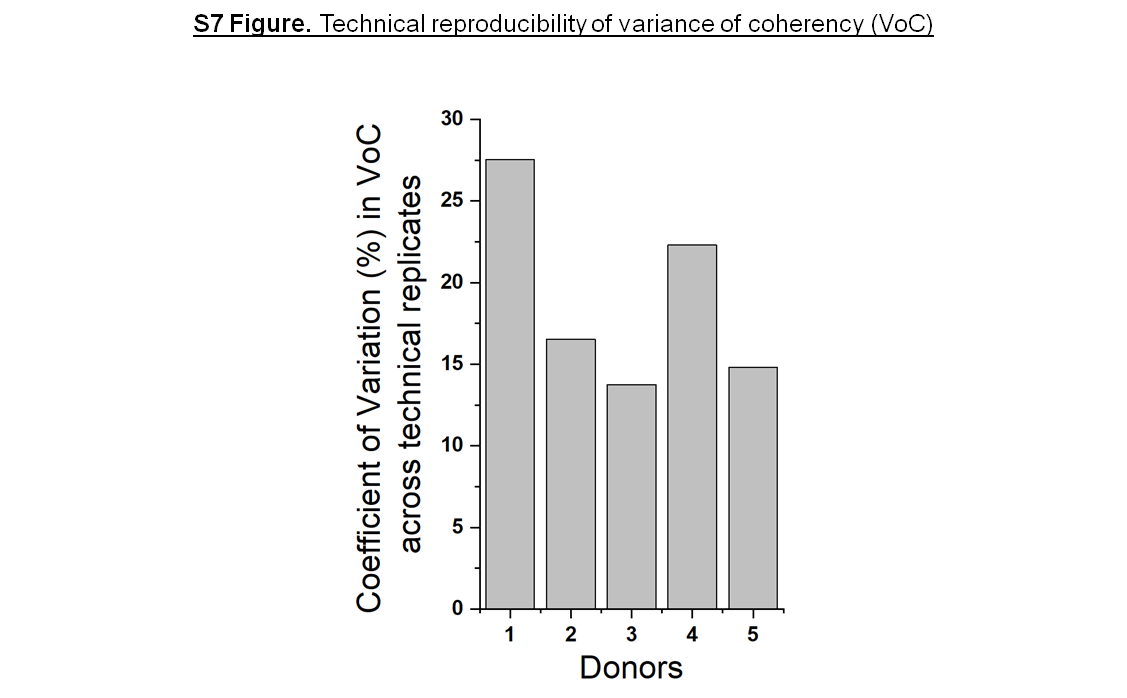

Supplement: S7 Fig — Coefficient of variation (%), computed as standard deviation / mean across 3 technical replicates for each of the 5 donors. (TIF) [file pone.0297769.s007.tif]

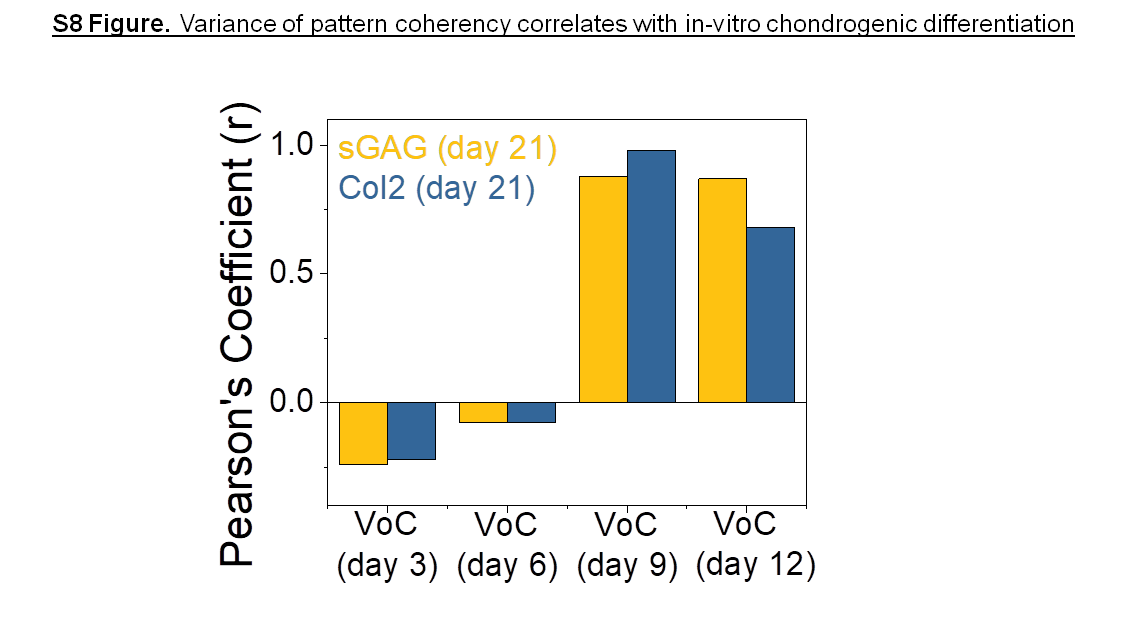

Supplement: S8 Fig — Pearson correlation coefficient for day 3, 6, 9, 12 VoC vs levels of matrix protein, n = 5 donors. (TIF) [file pone.0297769.s008.tif]

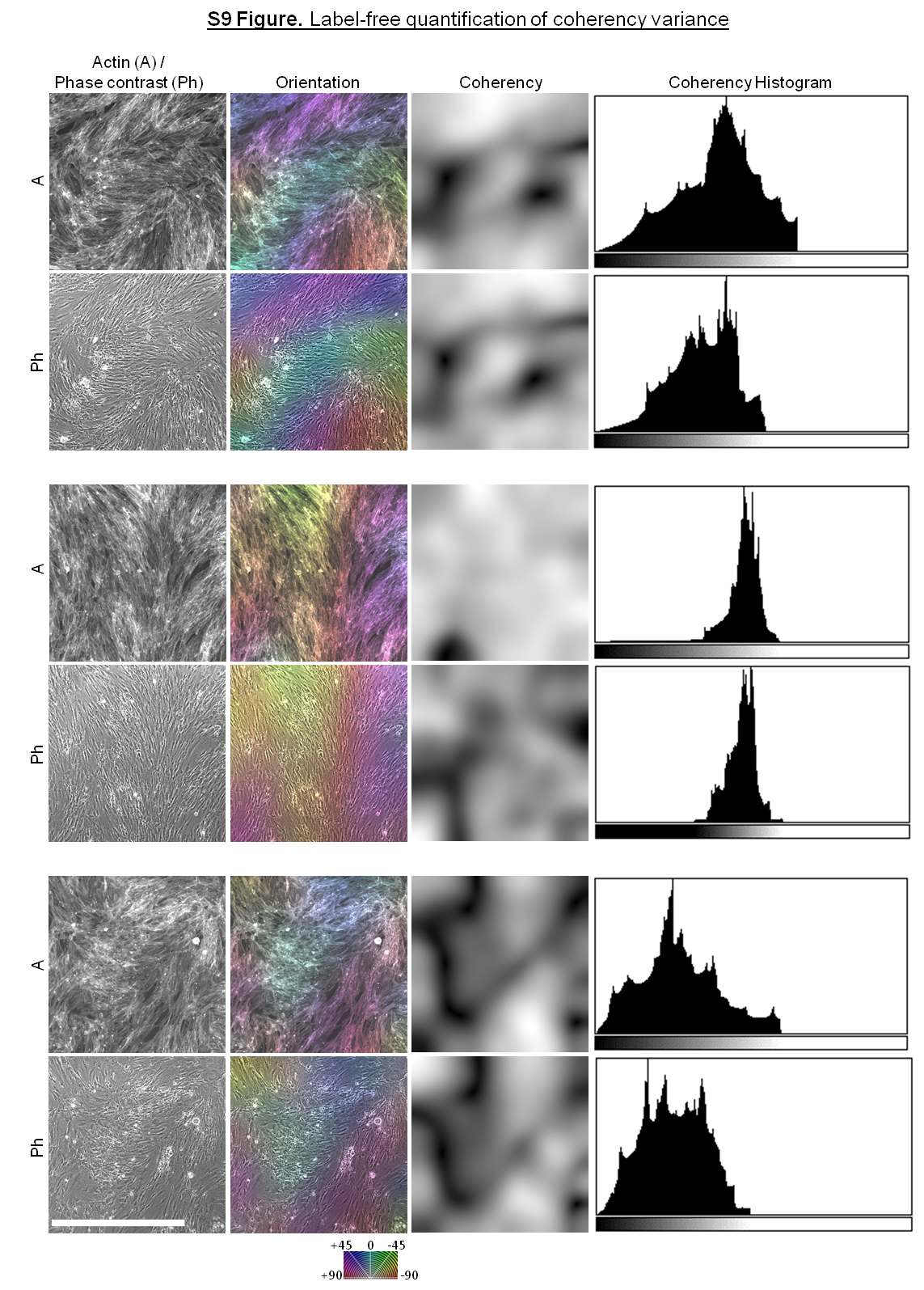

Supplement: S9 Fig — Comparison of orientation analysis for three different regions shows similarity in orientation, coherency and its variance from actin vs phase-contrast images. Scale bar 1000 μm. (TIF) [file pone.0297769.s009.tif]
